# The effect of adipose-derived stem cell sheets and CTGF on early flexor tendon healing in a canine model

**DOI:** 10.1038/s41598-018-29474-8

**Published:** 2018-07-23

**Authors:** Hua Shen, Rohith Jayaram, Susumu Yoneda, Stephen W. Linderman, Shelly E. Sakiyama-Elbert, Younan Xia, Richard H. Gelberman, Stavros Thomopoulos

**Affiliations:** 1 Department of Orthopaedic Surgery, Washington University, St. Louis, MO USA; 2 Department of Biomedical Engineering, Washington University, St. Louis, MO USA; 3 Department of Biomedical Engineering, University of Texas, Austin, TX USA; 4 Department of Biomedical Engineering, Georgia Institute of Technology, Atlanta, GA USA; 5 Department of Orthopedic Surgery, Department of Biomedical Engineering, Columbia University, New York, NY USA

## Abstract

Intrasynovial tendon injuries are among the most challenging in orthopedics. Despite significant improvements in operative and rehabilitation methods, functional outcomes continue to be limited by adhesions, gap formation, and rupture. Adhesions result from excessive inflammation, whereas tendon gapping and rupture result from inflammation-induced matrix degradation and insufficient regeneration. Therefore, this study used a combined treatment approach to modulate inflammation with adipose-derived mesenchymal stromal cells (ASCs) while stimulating tendon regeneration with connective tissue growth factor (CTGF). ASCs were applied to the repair surface via cell sheets and CTGF was delivered to the repair center via porous sutures. The effect of the combined treatment was assessed fourteen days after repair in a canine flexor tendon injury model. CTGF, either alone or with ASCs, reduced inflammatory (*IL1B* and *IL6*) and matrix degrading (*MMP3* and *MMP13*) gene expression, while increasing anti-inflammatory gene (*IL4*) expression and collagen synthesis compared to control repairs. The combined treatment was more effective than CTGF treatment alone, reducing the inflammatory *IFNG* and scar-associated *COL3A1* gene expression and increasing CD146^+^ tendon stem/progenitor cells at the tendon surface and interior along the core suture tracks. Therefore, the combined approach is promising in promoting early flexor tendon healing and worthy of further investigation.

## Introduction

Tendon injuries are common, affecting a large portion of the population and leading to physical impairment and large societal costs^1,2^. Many of these injuries are open wounds requiring extensive surgery, including complex mid-substance intra-synovial flexor tendon suturing methods^2–4^. Despite advances in operative techniques and rehabilitation methods, the outcomes of tendon repair are highly variable and result in a substantial clinical burden. Transected intrasynovial flexor tendons in the hand are particularly problematic, requiring operative repair and extensive rehabilitation in order to restore hand function. Although there have been significant improvements in tendon repair and rehabilitation, functional outcomes continue to be limited by adhesions, gap formation and rupture^2,4,5^. Adhesions result from excessive inflammation leading to matrix deposition between the repair surface and the surrounding sheath, whereas tendon gapping and rupture result from inflammation-induced matrix degradation and insufficient matrix regeneration to withstand applied loads at the repair site^6–9^.

Biologic therapies have the potential to improve these outcomes. Specifically, in prior studies, we demonstrated that adipose-derived mesenchymal stromal cells (ASCs) modulate tendon responses and facilitate regenerative healing via promotion of macrophage polarization toward the regenerative M2 phenotype and away from the default inflammatory M1 phenotype^10,11^. Furthermore, growth factors, such as bone morphogenetic protein 12 (BMP 12) and connective tissue growth factor (CTGF), have been shown to stimulate tendon regeneration by inducing exogenous and endogenous stem cell tenogenic differentiation^12–14^. However, due to the limited availability of effective, safe, and clinically feasible approaches for site-specific delivery of cells and growth factors to tendon, few studies have attempted to concurrently modulate inflammation and stimulate regeneration. Therefore, we established a biocompatible and tendon-specific system to deliver autologous ASCs to the tendon repair surface with cell sheets^10,11^ and growth factors to the interior of the tendon via porous sutures^15,16^. Using these innovative approaches, the current study investigated the effects of concurrent and site-specific applications of ASCs and CTGF on tendon healing using a clinically relevant canine flexor tendon injury and repair model^17,18^. It was hypothesized that ASCs and CTGF, by modulating inflammation and promoting matrix synthesis at the repair site, would enhance healing at 14 days after tendon repair.

## Results

### Porous sutures retain the mechanical properties of unmodified sutures and can deliver growth factor in a sustained manner

**Figure 1:**
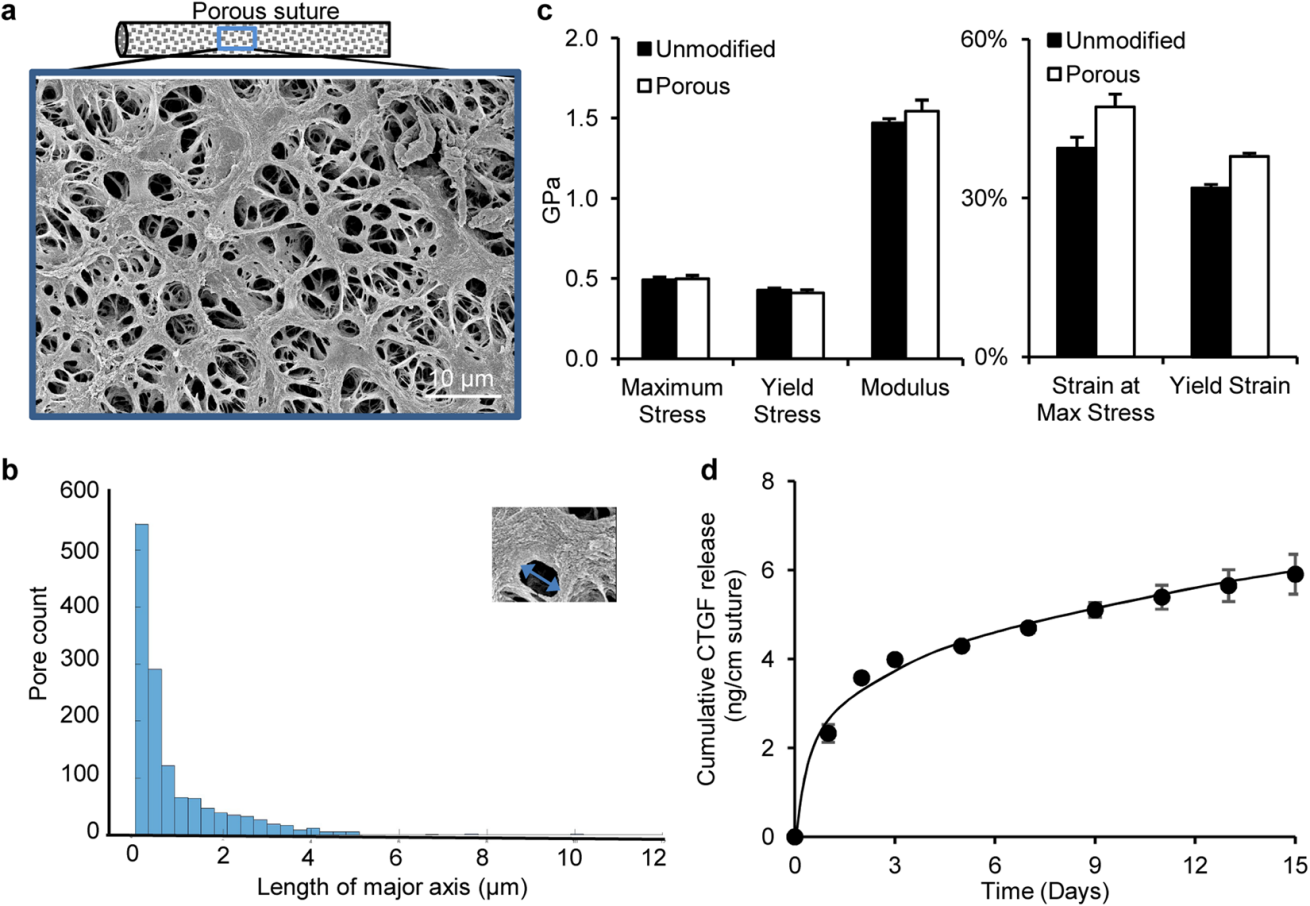
Structural, material, and delivery properties of porous sutures. (**a**) Representative scanning electromicroscopy image of the surface of a porous suture. (**b**) Pore size (the length of major axis) distribution of porous suture. (**c**) Mechanical properties of porous sutures compared to unmodified sutures. (**d**) Cumulative release of CTGF from porous sutures preloaded with 30 µg/mL CTGF.

To deliver CTGF to the interior of repaired tendons, 4-0 Supramid sutures were modified to create pores in their outer layer^15,16^. The resulting pores were visualized via scanning electron microscopy (Fig. 1a). Quantitative analysis revealed a mean pore area of 0.75 ± 1.64 µm^2^ and a mean length of the pore major axis of 0.98 ± 1.12 µm (Fig. 1b). Porous sutures exhibited mechanical properties comparable to unmodified sutures (Fig. 1c). To examine the capacity of the porous suture to deliver growth factor, CTGF (30 µg/mL) was loaded into porous sutures via a heparin/fibrin delivery system^19,20^. The release kinetics of CTGF from the loaded sutures was determined *in vitro* in three independent loading experiments. After an initial burst release over the first 3 days, CTGF was released at a steady rate of 0.08 ± 0.02 ng/day/cm suture during the subsequent 12 days (Fig. 1d). With a cumulative release of 5.9 ± 0.4 ng/cm suture over the full 15-day period and an average suture length of 20 cm/repair (Table 1), it is anticipated that approximately 120 ng of CTGF would be delivered to the repair center *in vivo*.

**Table 1:** A summary of animals involved in the study.

Group	Animal #	Age (year)	Weight (Kg)	Digit	Tendon usage	Adhesion	Gap (mm)/Rupture	Core suture length (cm)
Control	1	3.1	26.3	1-2nd	RNA	yes	no	20.3
	2	1.5	24.9	2-5th	RNA	no	1.8	20.9
	3	3.3	22.7	3-2nd	RNA	no	no	19.9
	4	1.7	24.9	4-5th	RNA	no	no	19.0
	5	3.1	22.2	5-2nd	RNA	yes	no	20.5
	6	2.0	24.0	6-5th	RNA	no	no	19.7
	7	1.8	23.6	7-2nd	histology	no	no	20.8
	8	2.9	20.4	8-5th	histology	no	no	21.0
	9	1.7	20.4	9-2nd	histology	no	no	19.3
	10	1.8	21.3	10-5th	excluded	n/a	rupture	19.9
CTGF	11	2.6	25.9	11-2nd	RNA	no	no	22.3
	12	2.5	24.0	12-5th	RNA	no	no	20.4
	13	1.5	22.7	13-2nd	RNA	yes	no	21.0
	14	2.0	22.7	14-5th	RNA	no	no	19.9
	15	2.0	20.4	15-2nd	excluded	n/a	rupture	19.9
	16	4.0	22.7	16-5th	histology	no	no	18.6
	17	4.5	25.4	17-5th	RNA	no	no	20.7
	18	1.5	23.1	18-2nd	RNA	no	no	20.5
	19	2.1	22.7	19-2nd	histology	no	no	20.6
	20	4.8	20.4	20-5th	histology	no	no	20.2
CTGF+ASC	11	2.6	25.9	11-5th	RNA	no	no	20.0
	12	2.5	24.0	12-2nd	RNA	no	no	20.3
	13	1.5	22.7	13-5th	RNA	yes	no	21.5
	14	2.0	22.7	14-2nd	RNA	no	no	20.4
	15	2.0	20.4	15-5th	RNA	no	no	20.1
	16	4.0	22.7	16-2nd	histology	no	no	20.6
	17	4.5	25.4	17-2nd	RNA	no	no	20.1
	18	1.5	23.1	18-5th	RNA	no	no	21.2
	19	2.1	22.7	19-5th	histology	no	no	19.9
	20	4.8	20.4	20-2nd	histology	no	no	20.2

### Site-specific delivery of ASCs and CTGF was achieved during tendon repair

**Figure 2:**
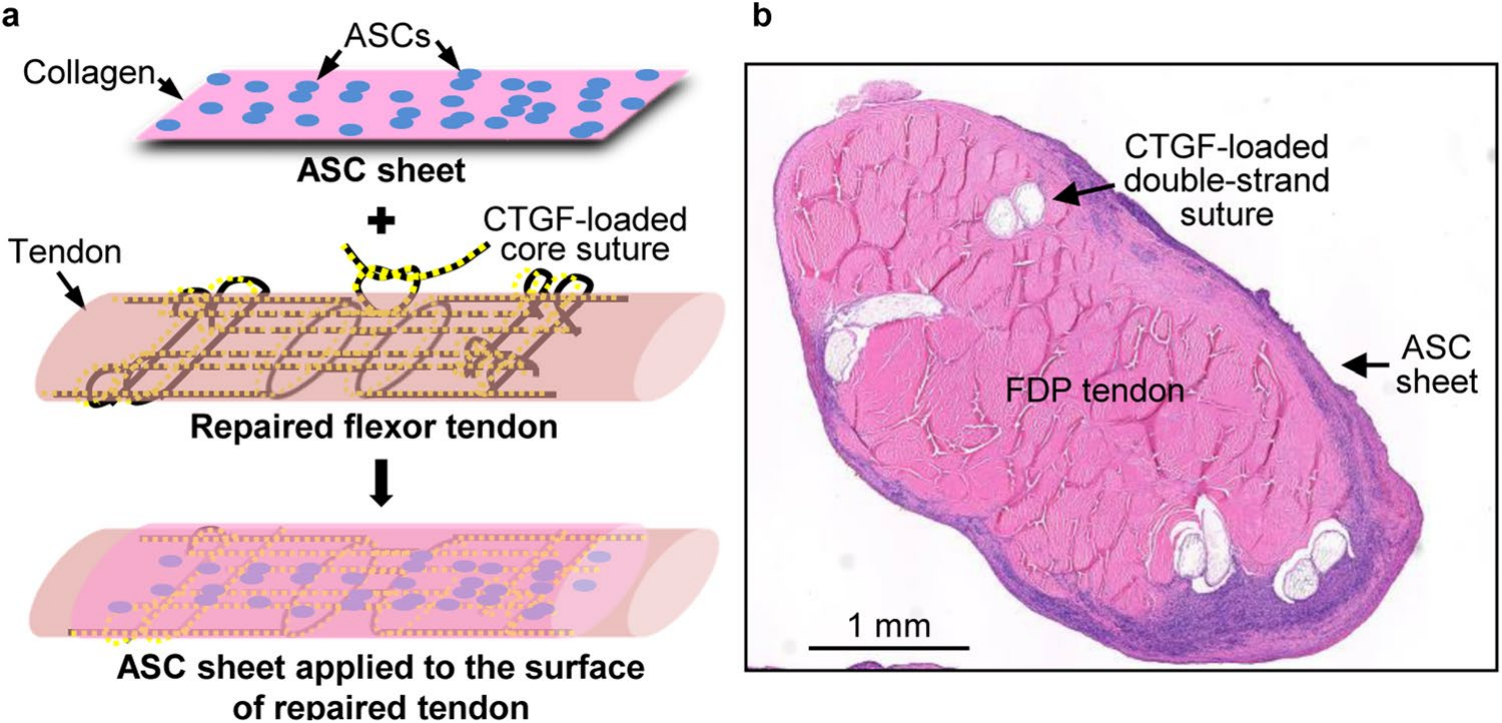
Site-specific delivery of ASCs and CTGF during flexor tendon repair. (**a**) A schematic illustration of CTGF and ASC delivery during flexor tendon repair. (**b**) A representative image of a hematoxylin and eosin-stained cross section of an CTGF+ASC treated canine flexor digitorum profundus (FDP) tendon 14 days after repair.

A clinically relevant canine flexor tendon transection and repair model was used to evaluate the potential of ASCs and CTGF for enhancing tendon healing^17,18^. As shown in Fig. 2, CTGF was delivered to the interior of repaired tendons using the porous core sutures described above, and ASCs were applied to the repair surface via our previously reported cell sheet approach^10,11^. The delivery of ASCs and growth factors was previously validated via tracking of GFP-expressing ASCs^10,11^ and pre-labeled proteins in porous sutures^15^ in repaired tendons. The biocompatibility of ASC sheet and porous suture was previously confirmed by cell viability assays *in vitro*^10,15^.

### The overall impact of ASCs and CTGF on early tendon healing response

The overall healing response was grossly compared among tendons from three repair groups (N = 10 per group): Control (repaired with unloaded porous suture), CTGF (repaired with CTGF-loaded porous suture) and CTGF+ASC (repaired with CTGF-loaded porous suture and ASC sheet) 14 days after flexor tendon repair. After sacrifice, repaired tendons were surgically exposed and visually assessed. As detailed in Table 1, the control group had the worst outcomes, with one rupture, one 1.8 mm gap, and two repairs with apparent adhesions. In contrast, only one rupture and one tendon with adhesions were noted in CTGF-treated tendons, and only one tendon with adhesions was observed in CTGF and ASC co-treated tendons. As such, the percentage of overall postoperative complication (adhesion plus gap/rupture) for Control, CTGF, and CTGF+ASC group were 40%, 20%, and 10%, respectively (“N-1” Chi-squared test, *P* = 0.342 and 0.131 for CTGF and CTGF+ASC vs. Control, respectively), implicating a potential positive effect of CTGF+ASC combined treatment in improving flexor tendon healing.

### ASCs and CTGF modulate the inflammatory response during tendon healing

**Figure 3:**
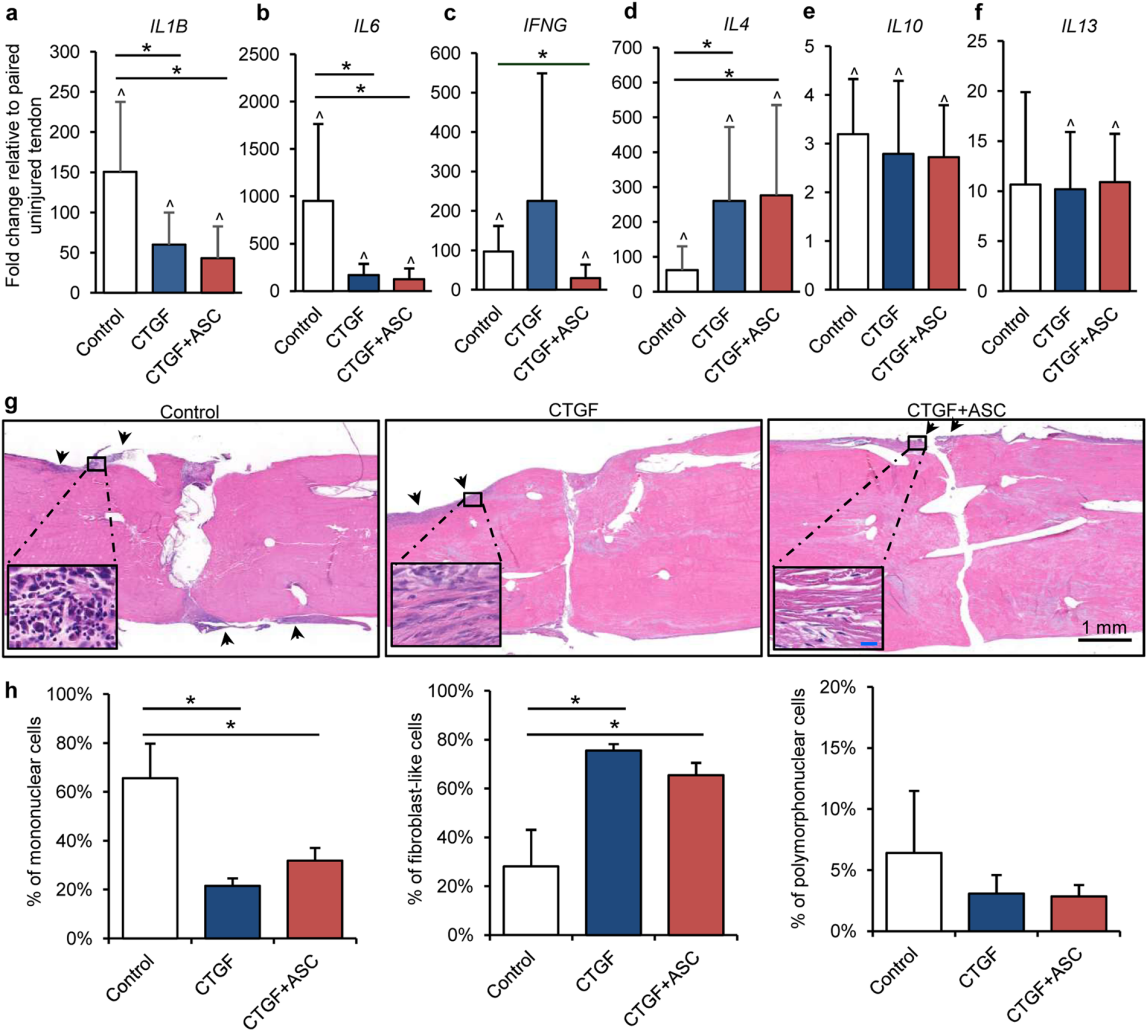
The impact of CTGF/CTGF+ASC treatment on tendon inflammatory response. (**a**–**f**) Changes in relative abundance of inflammation-related genes in flexor tendons 14 days after repair and indicated treatments. ^*^P < 0.05 between indicated groups by Dunn’s (b, c and d) or Student-Newman-Keuls post-hoc tests (**a**); ^^^*P* < 0.05 compared to paired uninjured tendons by paired t-tests. (**g** and **h**) Representative images (**g**) and quantification (**h**) of hematoxylin and eosin-stained coronal sections of zone 2 flexor digitorum profundus tendons subjected to the indicated treatments. The sections are ~250 µm from the volar surface of the tendons. The arrows indicate the regions where cells are accumulated at the tendon surface. The insets show enlarged views of the boxed region within each image (blue scale bar = 20 μm). ^*^P < 0.05 between indicated groups by Student-Newman-Keuls post-hoc tests.

ASCs are known to attenuate tendon inflammatory response by promoting macrophage polarization toward an alternative M2 phenotype and away from the default pro-inflammatory M1 phenotype^10,11^. CTGF, by enriching endogenous CD146^+^ tendon progenitor/stem cells, may produce a similar effect^13,21^. To determine the inflammation-related effects of ASCs and CTGF during tendon healing, gene expression levels were compared among repaired tendons from Control (N = 6), CTGF (N = 6) and CTGF+ASC (N = 7) groups. Tendon injury substantially increased the expression levels of pro-inflammatory genes *IL1B*, *IL6* and *IFNG* in control tendons (Fig. 3a–c, paired t-test compared to contralateral uninjured tendons, *P = *0.034, 0.013 and 0.013, for Control, CTGF, and CTGF+ASC groups, respectively). Treatment with CTGF, either applied alone or in combination with ASCs, significantly reduced *IL1B* (Fig. 3a; one-way ANOVA, *P* = 0.016) and *IL6* (Fig. 3b; one way-ANOVA, *P* = 0.002) expression relative to control tendons, thus demonstrating anti-inflammatory effects of CTGF. CTGF alone and in combination with ASCs enhanced *IL4* expression (Fig. 3d; one-way ANOVA, *P* = 0.039), a well-known M2 macrophage stimulator^22^, further supporting positive effects of treatment on inflammation during tendon healing. However, there were no significant differences when comparing CTGF to CTGF+ASC for the expression of most of the inflammation-related genes investigated, with the exception of *IFNG*, which was decreased in the CTGF+ASC group compared to control (Fig. 3c; one-way ANOVA, *P* = 0.014, Control vs. CTGF+ASC; *P* = 1.000, Control vs. CTGF). *IFNG* encodes Interferon γ, which primes the pro-inflammatory M1 macrophage phenotype^23^; the results therefore indicate an anti-inflammation function provided by ASCs. No apparent differences in *IL10* (Fig. 3e; one-way ANOVA, *P* = 0.771) and *IL13* (Fig. 3f; one-way ANOVA, *P* = 0.982) expression levels were detected between repair groups. Nevertheless, *IL10* was induced in all repaired tendons compared to paired uninjured tendons (Fig. 3e; paired t-test, *P* = 0.003, 0.001 and 0.000 for Control, CTGF, and CTGF+ASC, respectively). Furthermore, significant increases in *IL13* expression levels were detected in tendons from CTGF and CTGF+ASC but not Control groups compared to uninjured tendons (Fig. 3f; paired t-test, *P* = 0.138, 0.009 and 0.000 for Control, CTGF and CTGF+ASC, respectively).

At the tissue level, tendon samples from each repair group (N = 3 per group) were sectioned longitudinally and stained with hematoxylin and eosin. As shown in Fig. 3g, tendon injury induced cell accumulation (in dark blue) at the periphery of repaired tendons (arrows). Cell accumulation was more prominent in the control tendons than in the CTGF- or CTGF+ASC-treated tendons. A close examination and quantitative analysis of cellularity within these regions (insets in Fig. 3g,h) revealed distinct cell compositions between the control and treated tendons: the control tendons were primarily occupied by round mononuclear and polymorphonuclear cells (One-way ANOVA, *P* = 0.002); in contrast, more elongated fibroblast-like cells were present in CTGF- or CTGF+ASC-treated tendons (One-way ANOVA, *P* = 0.002). Collectively, these findings strongly support the gene expression results demonstrating anti-inflammatory effects of CTGF and CTGF+ASC during tendon healing.

### The effects of ASCs and CTGF on proliferation and tenogenesis during tendon healing

**Figure 4:**
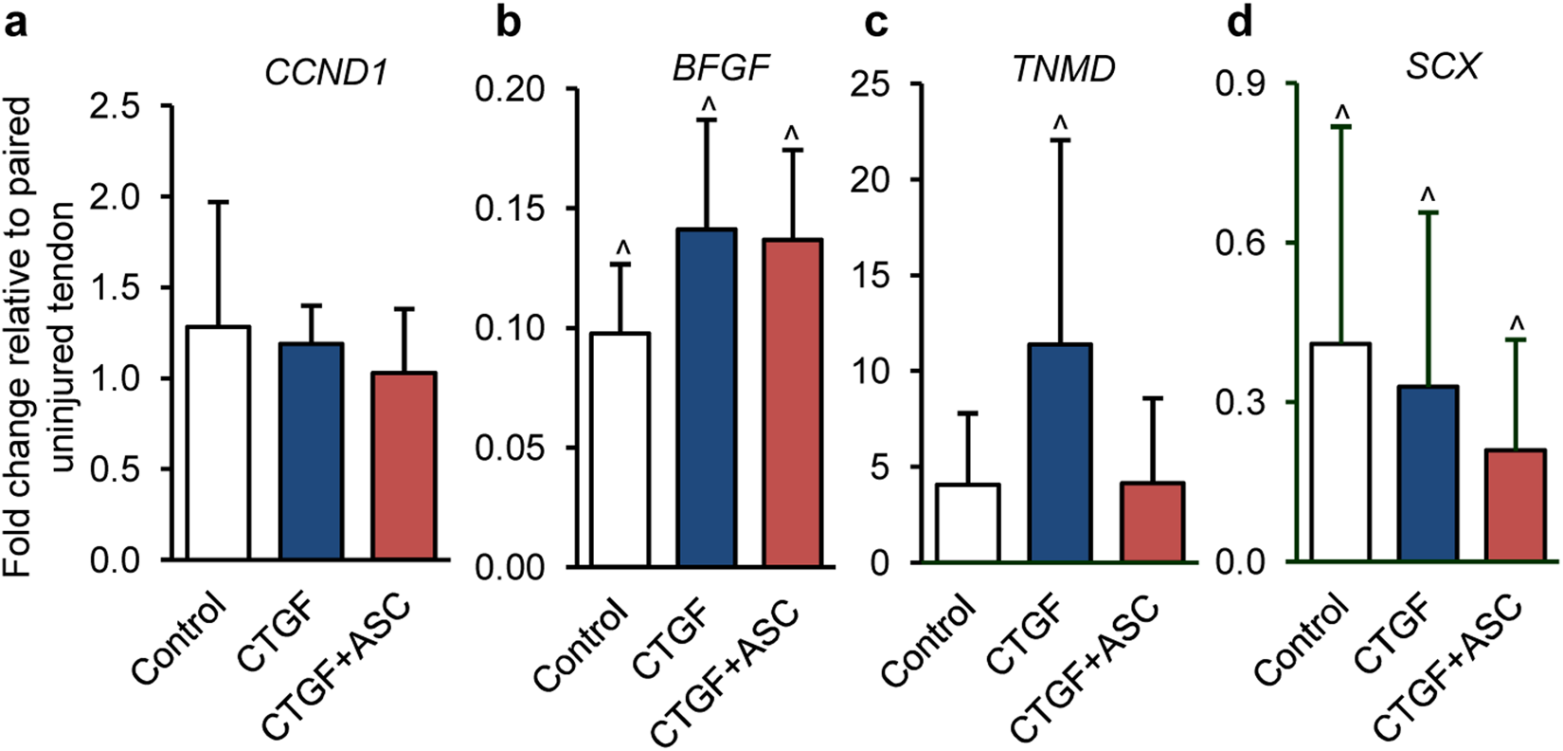
The impact of CTGF/CTGF+ASC treatment on tendon cell growth and differentiation. (**a**–**d**) Changes in relative abundance of genes involved in cell growth and differentiation in flexor tendons 14 days after repair and indicated treatments. ^^^*P* < 0.05 compared to paired uninjured tendons by paired t-tests.

The impact of ASC and CTGF on cell growth and differentiation was assessed at the mRNA level for genes involved in cell proliferation (*CCND1*, Fig. 4a) and fibroblast/tenocyte growth and differentiation (*BFGF*, *TNMD* and *SCX*, Fig. 4b–d). Compared to paired uninjured tendons, while all repaired tendons exhibited lower levels of *BFGF* (Fig. 4c; paired t-test, *P* < 0.001 for all three repair groups) and *SCX* (Fig. 4e; paired t-test, *P* = 0.023, 0.000 and 0.001 for Control, CTGF and CTGF+ASC group, respectively), only CTGF-treated tendons expressed a significantly higher than normal level of *TNMD* (Fig. 4d; paired t-test, *P* = 0.144 [Control], 0.014 [CTGF] and 0.050 [CTGF+ASC]), indicating a beneficial effect of CTGF treatment. No significant differences were detected when comparing the three repair groups for any gene examined (one-way ANOVA: *P* = 0.611 [*CCND1*], 0.078 [*BFGF*], 0.137 [*TNMD*] and 0.284 [*SCX*]).

**Figure 5:**
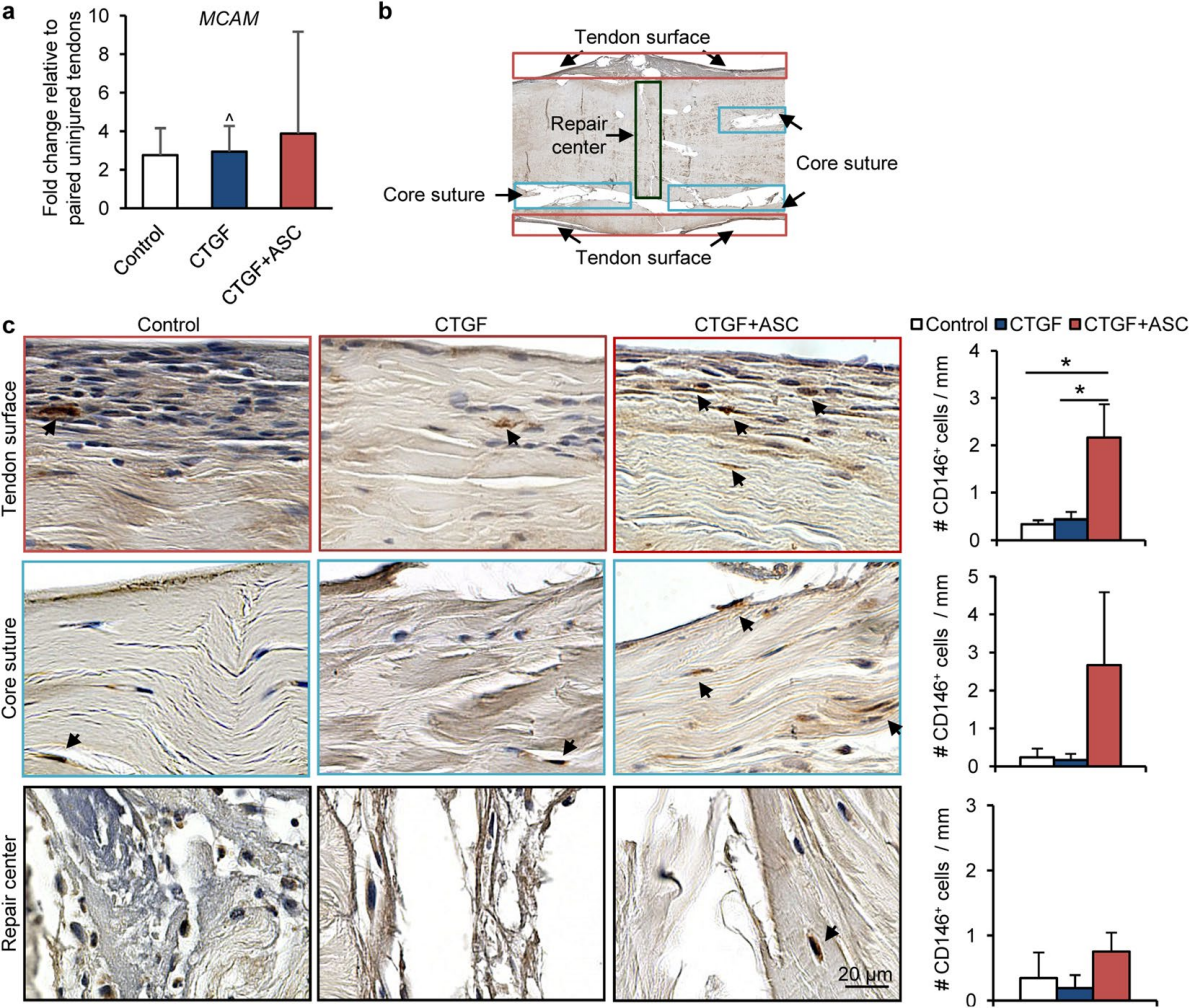
The impact of CTGF/CTGF+ASC treatment on CD146^+^ tendon progenitor/stem cell expression. (**a**) Changes in relative abundance of CD146 *gene MCAM* in flexor tendons 14 days after repair and indicated treatments. ^^^*P* < 0.05 compared to paired uninjured tendons by paired t-tests. (**b**) Annotated coronal section of a repaired flexor tendon indicating locations of regions of interest. (**c**) Representative images and quantifications of CD146 staining at the indicated regions of interest in flexor tendons from three repair groups. The sections are ~250 µm from the volar surface of the tendons. ^*^P < 0.05, by Student-Newman-Keuls post-hoc tests.

Previous reports have demonstrated induction of CD146^+^ stem cells by CTGF during tendon healing^14,21^. We therefore determined *MCAM* (encoding CD146) expression levels in repaired tendons. Regardless of the repair group, *MCAM* levels in repaired tendons were over two-fold greater than those in paired uninjured tendons (Fig. 5a; paired t-test, *P* = 0.075 [Control], 0.004 [CTGF] and 0.053 [CTGF+ASC]); however, no significant effect by either CTGF or CTGF+ASC treatment was detected (One-way ANOVA, *P* = 0.206). At the tissue level, CD146^+^ cells were detected via immunostaining on coronal sections of repaired tendons (N = 3 per group). Among the three regions of interest — tendon surface, core suture and repair center (Fig. 5b) — CD146^+^ cells (dark brown, arrows in Fig. 5c) mostly accumulated at the tendon surface away from the repair center (arrows in red boxes in Fig. 5b,c). CD146^+^ cells were also observed around the core suture (arrows in blue boxes in Fig. 5b,c) distal to the repair center (arrows in black boxes in Fig. 5b,c). Comparing the three repair groups, more CD146^+^ cells were detected at the tendon surface in CTGF+ASC-treated tendons than in Control and CTGF-treated tendons (One-way ANOVA, *P* = 0.003, Fig. 5c). A trending increase in CD146^+^ cells was also found in the region along core suture tracks in CTGF+ASC-treated tendons (One-way ANOVA, *P* = 0.05; Fig. 5c). No apparent difference in CD146+ cells at the repair center was noted in tendons between any of the repair groups (One-way ANOVA, *P* = 0.150; Fig. 5c).

### ASCs and CTGF promote matrix regeneration during tendon healing

**Figure 6:**
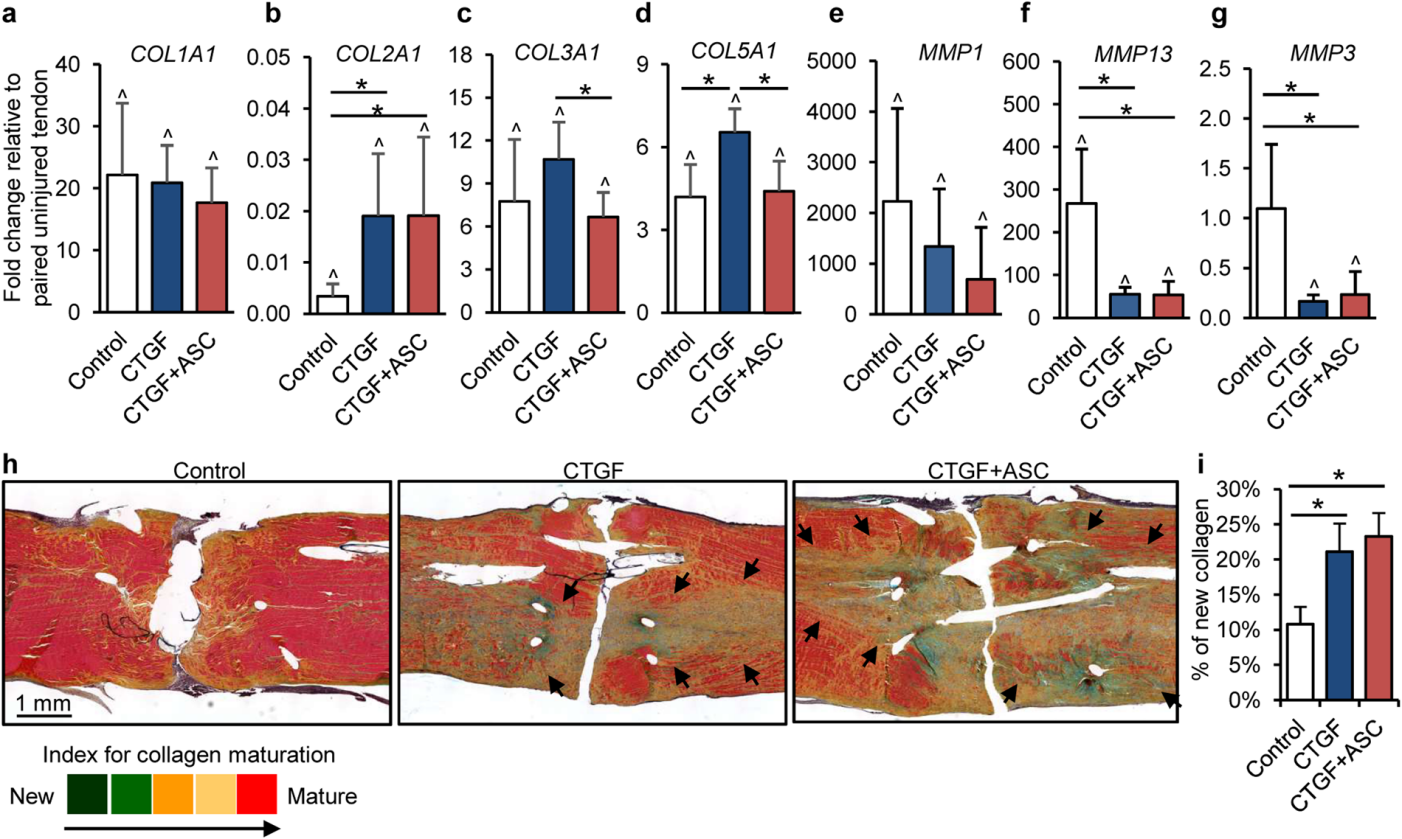
The impact of CTGF/CTGF+ASC treatment on tendon matrix remodeling. (**a**–**g**) Changes in relative abundance of genes associated with tendon matrix remodeling in flexor tendons 14 days after repair. ^*^P < 0.05 between indicated groups by Dunn’s (**b**,**f** and **g**) or Student-Newman-Keuls post-hoc tests (**c** and **d**). ^^^*P* < 0.05 compared to paired uninjured tendons by paired t-tests. (**h**,**i**) Representative images (**h**) and semiquantitative analysis (i) for new collagen synthesis in pentachrome-stained coronal sections of flexor tendons 14 days after repair and indicated treatments. The sections are ~250 µm from the volar surface of the tendons. The arrows point out the regions where newly formed collagens are present in treated tendons.

At the gene expression level, *COL1A1* was increased by ~20-fold in healing tendons compared to uninjured tendons (Fig. 6a; paired t-test, *P* < 0.001 compared to paired uninjured tendons for all three groups). In contrast, *COL2A1* expression in healing tendons was reduced by over 50-fold compared to uninjured tendons (Fig. 6b; paired t-test, *P* = 0.019 [Control], 0.014 [CTGF] and <0.001 [CTGF+ASC]). The reduction in *COL2A1* expression was attenuated following either CTGF or CTGF+ASC treatment (one-way ANOVA, *P* = 0.005). Expression of *COL3A1* and *COL5A1* was increased in healing tendons compared to uninjured tendons (Fig. 6c,d; paired t-test, *P* < 0.004 for all groups). Treatment with CTGF alone led to increased expression of *COL5A1* compared to control and CTGF+ASC groups (one-way ANOVA, *P* = 0.002 among three repair groups). The addition of ASCs to CTGF-treated tendons lowered *COL3A1* (encode type III collagen) level in repaired tendons (One-way ANOVA, *P* = 0.007 between CTGF and CTGF+ASC group). As type III collagen has been linked to scar formation and inferior tendon mechanical properties^24^, these results support a beneficial effect of the CTGF+ASC combined treatment.

With regard to matrix degrading enzymes, *MMP1* expression levels in repaired control tendons were increased by over 2000-fold compared to uninjured tendons (Fig. 6e; Signed Rank Test, *P* = 0.031). Likewise, *MMP13* levels in control tendons were over 200-fold greater than those in paired uninjured tendons (Fig. 6f; paired t-test, *P* = 0.006). Treatment either with CTGF or CTGF+ASC significantly attenuated the injury-induced *MMP13* expression in repaired tendons (Fig. 6f; one-way ANOVA, *P* = 0.004 among three repair groups). Although no significant change in *MMP1* expression was detected following CTGF or CTGF+ASC treatment (Fig. 6e; one-way ANOVA, *P* = 0.159), a trend toward reduced *MMP1* expression was apparent in treated tendons. In addition, *MMP3* levels, which were not altered in untreated control tendons (Fig. 6g; paired t-test, *P* = 0.926 vs. paired uninjured tendons), were reduced in tendons treated with either CTGF or CTGF+ASC (Fig. 6g; one-way ANOVA, *P* = 0.009 among three repair groups). Collectively, gene expression analysis indicated that CTGF and CTGF+ASC treatments enhanced the anabolic response while inhibiting the catabolic response associated with tendon matrix remodeling.

At the tissue level, pentachrome staining was performed to assess tendon matrix remodeling in repaired tendons (N = 3 per group). Pentachrome staining is indicative of the stage of collagen maturation according to a color scheme from green (immature) through red (mature) (Fig. 6h)^25,26^. Robust collagen production was seen in repaired tendons, particularly following CTGF- or CTGF+ASC treatment (Fig. 6h). Semiquantitative analysis confirmed that newly produced collagens occupied approximately 10% greater area in CTGF and CTGF+ASC treated tendons than in control tendons (Fig. 6i; One-way ANOVA, *P* = 0.008 among three groups). Moreover, the newly produced collagen was present in the tendon interior (black arrows in Fig. 6h) and largely in parallel to the tracks of the core suture, supporting an effect of suture-delivered CTGF.

## Discussion

After operative repair, tendon healing progresses through phases of inflammation, proliferation, and remodeling. Each of these phases is a potential therapeutic target for enhanced healing. Previously, we demonstrated positive effects from ASC administration on the early inflammatory phase of healing and positive effects from CTGF application on the later proliferative phase of healing. These treatments were combined in the current study: ASCs were delivered to the flexor tendon surface via cell sheets and CTGF was delivered to the tendon interior via porous sutures. The approach was first validated by demonstrating the mechanical integrity and delivery capability of the porous sutures. Its efficacy was then demonstrated in a clinically relevant large animal model of flexor tendon injury and repair. At the mRNA and tissue levels, CTGF+ASC treatment led to an attenuated tendon inflammatory response and increased tendon matrix regeneration, thus providing a promising new treatment strategy for flexor tendon repair.

CTGF was introduced into this study due to its potential to promote tendon regeneration^13,14^. Interestingly, in addition to enhancing *TNMD* expression and tendon matrix synthesis, CTGF was found to attenuate tendon inflammation, leading to reduced *IL1B* and *IL6* expression and inflammatory cell infiltration during flexor tendon healing. As the effect was accompanied by an increased expression of a M2 stimulator gene *IL4* and a switch of primary infiltrated cells from inflammatory mononuclear cells to fibroblast-like cells, CTGF might be involved in modulating macrophage polarization. This concept is consistent with a recent report showing CTGF can suppress iNOS^+^ M1 macrophages via enrichment of CD146^+^ tendon stem/progenitor cells^21^. However, when assessing the expression level of the CD146 gene, *MCAM*, and the immunohistochemical pattern of CD146^+^ cells in repaired tendons, we found no apparent differences between CTGF-treated and untreated control tendons. This discrepancy may be due to the 14-day time point studied, as the presence of CTGF-enriched CD146^+^ cells typically occurs early in tendon healing. Specifically, these cells have been shown to peak within the first week after CTGF delivery and are barely detectable by two weeks^14^. Moreover, as CD146^+^ cells account for less than 1% of all cells in uninjured tendon^14^, the effect of CTGF might also be limited by a lack of endogenous stem cells within flexor tendon. In line with this premise, the current study demonstrates an enrichment of CD146^+^ cells in the CTGF-repaired tendons within the treatment group that included ASCs. Alternatively, other mechanisms may contribute to the anti-inflammation function of CTGF. A follow-up short-term study (e.g., 7 days after repair) would be helpful to assess the potential causal link between CD146^+^ cells and CTGF function and to decipher the underlying mechanism by which CTGF modulates inflammation during flexor tendon healing.

As expected, the combined application of ASCs and CTGF was more effective than CTGF treatment alone in regulating tendon inflammation and regeneration. In accord with the roles of ASCs in modulating tendon inflammation and promoting CD146^+^ tendon stem or progenitor cells after tendon repair^10,11^, the combined treatment reduced the expression of *IL1B, IL6, IFNG* (inducing inflammatory M1 macrophage phenotype), and *COL3A1* (associated with scarring) and simultaneously increased CD146^+^ cells in repaired tendons. Accordingly, the combined treatment led to improved collagen production and a trending reduction of postoperative complications. Future studies should evaluate an ASC-alone treatment group and determine macrophage polarization due to ASC, CTGF or CTGF+ASC treatment. These studies may further differentiate the roles and mechanisms of ASCs, CTGF and CTGF+ASC in modulating tendon inflammation and healing responses.

A limitation of this study was the examination of only one time-point of healing. In addition to assessing the short-term effect, as discussed above, analysis of longer healing time-points are also necessary to determine if the early changes observed here will lead to improved biomechanical outcomes. Nevertheless, this study demonstrates the feasibility of cell sheets and porous suture as vehicles for the site-specific delivery of cells and growth factors in flexor tendon repair. Furthermore, the results support the use of ASCs and CTGF as a combined strategy for enhancing flexor tendon healing and is worthy of further investigation.

## Methods

### Study Design

With the approval from Washington University Institutional Animal Care and Use Committee, 10 adult female canines were included in this study. All experiments were performed in accordance with relevant guidelines and regulations. As detailed in Table 1, with a paired design, zone 2 flexor digitorum profundus tendon (FDP) transections and repairs were conducted in the 2^nd^ and 5^th^ digits of the right front paw of each animal. Specifically, FDP tendons were transected at the level of the proximal interphalangeal joint and repaired using an 8-strand Winters-Gelberman core suture technique^27^. The repaired tendons were assigned to two treatment groups: CTGF and CTGF+ASC (N = 10/group). In the CTGF group, the repaired digits were treated with porous suture loaded with CTGF. In the CTGF+ASC group, in addition to CTGF, the repaired tendons were further treated with a thin cell sheet containing autologous ASCs^10^. In order to minimize animal usage, flexor tendons from a separate study^16^ (N = 10) were repaired in the same manner as those from the CTGF and CTGF+ASC groups with porous suture and without CTGF and used as control. The corresponding left (non-operated) digital flexor tendons served as uninjured controls (Uninjured group). With 10 tendon samples from each group, 7 samples were designated for RNA isolation and subsequent tendon gene expression analysis, and the remaining 3 samples were assigned for histological assessments. All repaired limbs were immobilized after surgery using fiberglass shoulder spica casts with the elbows flexed to 90° and the wrists flexed to 70° and subjected to controlled passive mobilization as detailed previously^9^.

### Generation and characterization of CTGF-loaded porous sutures

4-0 Supramid sutures (S. Jackson, Alexandria, VA) were pre-treated with a swelling and freeze-drying technique to create porous at the outer layer of the suture^15^. The porosity of sutures was determined by scanning electron microscopy and analyzed via MATLAB software using a custom-made code. Uniaxial tensile testing was performed to compare material properties between porous and unmodified sutures (N = 7–8/group) as previously described^16^.

Recombinant human CTGF (BioVendor, Asheville, NC) was loaded into porous sutures via a heparin/fibrin delivery system (HBDS)^19,20^. In brief, dry sterile sutures were first incubated in a loading solution containing 58.8 µM plasminogen-depleted human fibrinogen (EMD Millipore, Burlington, MA), 423 µM bi-domain HBDS peptide (GenScript, Piscataway, NJ), 106 µM heparin (Sigma Aldrich, St. Louis, MO) and 30 µg/ml CTGF in TBS/BSA (50 mM Tris [pH 7.4], 150 mM NaCl and 0.1% w/v BSA) at 4 °C overnight. The loaded sutures were then incubated in a thrombin solution containing 20 U/ml thrombin (Sigma Aldrich), 13.7 mM CaCl_2_ and 30 µg/ml CTGF in TBS/BSA at 37 °C for 2 hours. CTGF release from loaded porous sutures (N = 3) was determined in TBS/BSA solution at 37 °C. The solution was replaced with fresh TBS/BSA daily in the first three days after loading and then every other day until 15 days after loading. CTGF contents in the replaced TBS/BSA solutions were determined with a human CTGF ELISA kit (Bio Ocean, Shoreview, MN) using a protein standard generated with the CTGF from BioVendor.

### *In vivo* delivery of ASC sheets

Two weeks prior to tendon repair, autologous ASCs were isolated from subcutaneous fat tissues and expanded in culture as described previously^10,11^. Passage 3 ASCs were subsequently cultured on the collagen sheet at a density of 16,000 cells/cm^2^ for 3–4 days *in vitro* and then applied *in vivo* following suture repair as illustrated in Fig. 2a.

### Gene expression analysis

Fourteen days after repair, tendon fragments flanking the repair site (approximately 10 mm from each side of the transection line) were dissected and subjected to total RNA isolation, cDNA synthesis, and quantitative TaqMan RT-PCR as described previously^10,11^. All TaqMan primers and probes used in this study were purchased from Applied Biosystems TaqMan Gene Expression Assays (Woolston, UK; Table 2) except for *SCX*, which was custom designed (gene name, scleraxis; accession number, XM_005628297; forward primer, 5′-gca agc tct cca aga tcg ag-3′; reverse primer, 5′-ctt tct ctg gtt gct gag gc-3′; probe, 5′-gtc cag cta cat ctc gca cc-3′). *GAPDH* and *PPIB* were used as endogenous reference genes. The relative abundances of target genes in each sample were determined with ∆∆Ct method and expressed as fold changes of respective uninjured controls.

**Table 2:** Commercial Taqman primers and probes used in the study.

Gene symbol	Gene name	Assay number	Accession number	Amplicon length (bp)
*BFGF*	basic fibroblast growth factor	Cf03460065_g1	XM_003432481.3	147
*CCND1*	cyclin D1	Cf02626707_m1	NM_001005757.1	85
*COL1A1*	collagen, type I, alpha 1	Cf02623126_m1	NM_001003090.1	87
*COL2A1*	collagen, type II, alpha 1	Cf02622836_m1	NM_001006951.1	72
*COL3A1*	collagen, type III, alpha 1	Cf02631370_m1	XM_845916.4	78
*COL5A1*	collagen, type V, alpha 1	Cf02645008_m1	XM_014116870.1	77
*GAPDH*	glyceraldehyde-3-phosphate dehydrogenase	Cf04419463_gH	NM_001003142.2	54
*IFNG*	interferon, gamma	Cf02623316_m1	NM_001003174.1	117
*IL1B*	interleukin 1, beta	Cf02671953_g1	NM_001037971.1	78
*IL4*	interleukin 4	Cf02623112_m1	NM_001003159.1	97
*IL6*	interleukin 6	Cf02624152_g1	NM_001003301.1	80
*IL10*	interleukin 10	Cf02624265_m1	XM_001003077.1	89
*IL13*	interleukin 13	Cf02624081_m1	NM_001003384.1	95
*MCAM*	melanoma cell adhesion molecule/CD146	Cf02651439_m1	XM_014113367.1	64
*MMP1*	matrix metallopeptidase 1	Cf02651000_g1	XM_546546.5	63
*MMP3*	matrix metallopeptidase 3	Cf02625960_m1	NM_001002967.1	79
*MMP13*	matrix metallopeptidase 13	Cf02623587_m1	XM_536598.4	78
*PPIB*	peptidylprolyl isomerase B (cyclophilin B)	Cf02629556_m1	XM_847296.4	78
*TNMD*	Tenomodulin	Cf02665570_m1	XM_538101.4	106

### Tendon histology and immunohistochemistry

Serial coronal paraffin sections (5 µm thick) were prepared from flexor tendons as previously described^10,11^. After deparaffinization, sections were either subjected to hematoxylin and eosin or pentachrome staining (American MasterTech, Lodi, CA)^25,26^ to examine the overall healing response and collagen regeneration, respectively. The cellularity at the tendon surface was determined by counting the number of fibroblast-like cells, polymorphonuclear cells, and mononuclear cells on hematoxylin and eosin-stained slides at a magnification of 40 at the predefined region indicated in Fig. 5b. The results are shown as the percentage of total cell counts for each cell type. The amount of new collagen formation was assessed by determining tissue area and mature collagen area (in red) in pentachrome stained sections using Adobe Photoshop 12.1 (Adobe Systems Incorporated) and expressed as a percentage of new collagen area in the entire tendon section: (tissue area - mature collagen area)/tissue area × 100%. For CD146 immunohistochemistry, after heat-induced antigen retrieval, sections were incubated with rabbit anti-CD146 antibodies (Abcam, Cambridge, UK; #Ab75769, 1:200 dilution) at 4 °C overnight. After three washes with 0.05% Triton X-100 in PBS (PBST), the sections were further incubated with biotinylated anti-rabbit IgG (Sigma Aldrich; 1:400 dilution) at room temperature for 1 hour, followed by ExtrAvidin-Peroxidase (Sigma Aldrich; 1:100 dilution) for 30 min. After three washes with PBST, the sections were developed with a DAB-Plus Substrate Kit (Life Technologies, Carlsbad, CA) and then counterstained with Mayer’s hematoxylin. CD146^+^ cells at the regions of tendon surface, core suture, and repair center (Fig. 5b) were counted and the results were normalized by the lengths of tendon surface, core suture, and transection line, respectively on coronal sections of repaired tendons covering a region approximately 10 mm from each side of the transection line.

### Statistics

Unless described elsewhere, all data are shown as mean + standard deviation; a one-way analysis of variance (ANOVA) followed by Student-Newman-Keuls or Dunn’s post-hoc test (when appropriate) was performed to compare gene expression among groups; two-tailed paired Student’s t-tests were used to compare gene expression levels in repaired and contralateral uninjured tendons; a “N−1” Chi-squared test was employed to compare the percentage of postoperative complications between treated and control groups^28,29^. The significance level was set at *P* < 0.05.
